# Possible Impact of Salivary Influence on Cytokine Analysis in Exhaled Breath Condensate

**Published:** 2007-10-12

**Authors:** T. Ichikawa, K. Matsunaga, Y. Minakata, S. Yanagisawa, K. Ueshima, K. Akamatsu, T. Hirano, M. Nakanishi, H. Sugiura, T. Yamagata, M. Ichinose

**Affiliations:** The Third Department of Internal Medicine, Wakayama Medical University, School of Medicine, Wakayama, Japan

**Keywords:** asthma, cytokine, chemokine, growth factor, epithelial lining fluid

## Abstract

**Background:**

Exhaled breath condensate (EBC) is thought to contain substances of the lower airway epithelial lining fluid (ELF) aerosolized by turbulent flow. However, contamination by saliva may affect the EBC when collected orally.

**Objective:**

The purpose of this study was to compare the cytokine expression levels in EBC with those in saliva, and to clarify the influence of saliva on cytokine measurements of EBC.

**Methods:**

EBC and saliva samples were obtained from 10 adult subjects with stable asthma. To estimate differences in the contents of substances between EBC and saliva, the total protein concentration of each sample was measured. Further, we also measured the total protein concentration of ELF obtained from another patient group with suspected lung cancer using a micro sampling probe during bronchoscopic examination and roughly estimated the dilution of EBC by comparing the total protein concentration of EBC and ELF from those two patient groups. The cytokine expression levels of EBC and saliva from asthmatic group were assessed by a cytokine protein array.

**Results:**

The mean total protein concentrations in EBC, saliva and ELF were 4.6 μg/ml, 2,398 μg/ml and 14,111 μg/ml, respectively. The dilution of EBC could be estimated as 1:3000. Forty cytokines were analyzed by a cytokine protein array and each cytokine expression level of EBC was found to be different from that of saliva. Corrected by the total protein concentration, all cytokine expression levels of EBC were significantly higher than those of saliva.

**Conclusion:**

These results suggest that the salivary influence on the cytokine assessment in EBC may be negligible.

## Introduction

Exhaled breath condensate (EBC) is collected by cooling exhaled air. Collecting EBC is a noninvasive and repeatable method, and many researchers have reported the usefulness of EBC for measuring airway inflammatory molecules in respiratory diseases such as asthma, chronic obstructive pulmonary disease (COPD) and cystic fibrosis (Kharitonov and Barnes, 2004; Liu, J and Thomas, 2005; Barnes et al. 2006). EBC potentially reflects the volatile and non-volatile substances derived from all the airways. Non-volatile substances in airway epithelial lining fluid (ELF) are thought to be aerosolized by the turbulent flow generated mainly in the lower airway. Then, they are diluted by exhaled water vapor and incorporated into EBC. The dilution of EBC has been estimated using the conductivity, total cations and urea of plasma and EBC (Effros et al. 2004). When EBC is collected orally, it could contain substances derived from saliva (Silkoff et al. 2006). Some solutes measured in EBC such as amylase, and eicosanoids have been examined as markers of salivary contamination (Kharitonov and Barnes, 2001; Griese et al. 2002). The EBC pH from COPD patients has been reported to be unsuitable as a marker of airway acidification because it is affected by the volatile salivary acid (Effros et al. 2006). Leukotrien B4 in EBC has been reported to be derived from saliva (Gaber et al. 2006). However, the proportional contribution of saliva on the cytokine assessment of EBC has not yet been sufficiently studied.

Several molecules such as eicosanoids, chemokines and cytokines have been measured in EBC with the use of various assays such as a specific enzyme immunoassay (Shahid et al. 2002; Ko et al. 2006), gas chromatography/mass spectrometry (Cap et al. 2004), and cytometry beads array (Sack, U et al. 2006, Gessner et al. 2005). However, the precise molecular properties in EBC have not been clearly determined because the concentration of molecules measured in EBC is extremely low and there is relatively high variability in repeated measurements (Horvath et al. 2005; Hunt, 2002). We previously reported that a cytokine array assay was useful for cytokine analysis of EBC obtained from asthmatic patients (Matsunaga et al. 2006). Although this was a semiquantitative assay, the expression levels of forty cytokines could be simultaneously measured and we found that several cytokines, such as interleukin (IL)-4, IL-8, IL-17, tumor necrosis factor-alpha (TNF-α), regulated upon activation, normal T-cell expressed (RANTES), interferon (IFN)-γ-inducible protein 10 (IP-10), transforming growth factor-beta 1 (TGF-β1), macrophage inflammatory protein 1 alpha (MIP-1α) and MIP-1β were more up-regulated in EBC from asthmatic patients than in that from healthy controls (Matsunaga et al. 2006). However, the concern about the salivary influence on the results remains.

In the present study, we compared the cytokine expression levels in EBC with those in saliva to evaluate the influence of saliva on the cytokine measurements of EBC. Furthermore, we estimated the extent to which the contents in ELF and saliva contribute to EBC by comparing the total protein concentration of EBC and saliva from an asthmatic group with that of ELF from another patient group who had undergone bronchoscopic examination.

## Materials and Methods

### Study subjects

For EBC and saliva collection, adult patients with stable asthma were recruited. All patients met the American Thoracic Society criteria for asthma (The official statement of the American Thoracic Society, 1986). Stable asthma was defined as an absence of unscheduled physician visits for asthma care, unchanged use of asthma medication for maintenance therapy, and stable use of rescue medication for at least 4 weeks before sample collection. The patients who used regular anti-asthmatic medications except inhaled corticosteroids, had oral diseases or were current smokers were excluded. Patients planning to have a bronchoscopic examination were recruited for ELF collection. Patients with suspected infectious disease or high risk of bleeding were excluded. This study was approved by the ethics committee of Wakayama Medical University and all patients gave written informed consent.

Ten patients were recruited for each subject group. Among the asthmatic patients, seven required daily therapy with inhaled corticosteroids equivalent to a dose of 400 μg or more fluticasone. Two patients were ex-smokers. Two patients had a hypertension and used antihypertensive drugs. Among the patients for ELF collection, four were current smokers and four patients were ex-smokers. Two patients had stage II COPD without medication. Two patients had hypertension and one patient used antihypertensive drugs. After bronchoscopic examinations, seven patients were diagnosed as having lung cancer (three patients as adenocarcinoma, three patients as non-small cell lung carcinoma, one patient as metastatic lung carcinoma) and three patients exhibited no abnormal findings. The characteristics of the study subjects are listed in Table 1.

### Study design

This study was cross-sectional. Subjects with asthma attended the outpatient clinic at the Wakayama Medical University Hospital on one occasion for clinical examination, spirometry, and collection of both EBC and saliva samples. Subjects planning to have a bronchoscopic examination were admitted to the hospital and underwent spirometry and bronchoscopic examination. Spirometry was performed the day before the broncoscopic examination.

### EBC, saliva and ELF collection

The EBC was collected by using a condenser, which permitted noninvasive collection of condensed exhaled air by freezing it to −20 °C (Ecoscreen; Jaeger, Hoechberg, Germany) (Montuschi et al. 2000). The subjects breathed through a mouthpiece and a two-way non-rebreathing valve, which also served as a saliva trap. Subjects were asked to breath at a normal frequency and tidal volume, wearing a nose-clip, for 20–30 minutes. The collected EBC was melted and transferred to 1 ml Eppendorf tubes and immediately stored at −80 °C until investigation. The mean volume collected was approximately 2–3 ml.

Saliva was collected by expectoration into plastic tubes following EBC collection. Collected saliva samples were centrifuged at 300 g for 5 minutes and the separated supernatant was stored at −80 °C until investigation.

ELF samples were collected using bronchoscopy with microsampling probes (model BC-402C; Olympus, Tokyo, Japan) as previously described (Yamazaki et al. 2003). Briefly, the probe comprised a 2.5 mm outer diameter polyethylene sheath and an inner 1.9 mm-polyester fiber rod probe attached to a stainless steel guide wire. This probe immediately absorbs fluid. A flexible fiberoptic bronchoscope (model BF-P240; Olympus) was inserted into the right or left main bronchus after local anesthesia of the upper respiratory tract was achieved with a few milliliters of 2–4% lidocaine. After the channel of the bronchofiberscope was flushed with air, the microsampling probe was inserted through the channel into a main bronchus. Then, the inner probe was advanced slowly into the airway, and sampling of ELF was performed by placing the probe gently at a site of the targeted bronchial wall for 10 seconds. To avoid blood contamination, the subject was asked to hold a breath while the inner probe was placed at the bronchus. The inner probe was withdrawn into the outer tube, and both devices were withdrawn simultaneously. If visible blood contamination on the inner probe was detected, we inserted a microsampling probe again and recollected the ELF sample. The wet inner probe was cut 2cm from its tip. Three collected tips were placed in a tube and centrifuged at 10,000g for 10min. The solution was transferred to a new tube and stored at −80 °C until investigation. The probe was the dried and weighed again to measure the ELF volume recovered.

Cytokine assessment and detection of the protein concentration were performed within 4 weeks after collection of the EBC, saliva and ELF samples.

### Cytokine measurements

Human Inflammation Antibody III (Ray Biotech Inc., Norcross, Ga, U.S.A.), consisting of 40 different cytokine and chemokine antibodies spotted in duplicate onto a membrane, was utilized (Huang, 2001a; Huang, 2001b; Lin et al. 2003; Turtinen et al. 2004). Briefly, the membranes were blocked with 10% bovine serum albumin in Tris-buffered saline, and then 1.0 ml each of EBC and saliva obtained from the asthmatic subjects was added to each membrane and incubated at room temperature for 2 hours. The membranes were washed, and 1.0 ml of primary biotin-conjugated antibody was added and incubated at room temperature for 2 hours. Following a thorough wash, the membranes were incubated with 2.0 ml of horseradish peroxidase (HRP)-conjugated streptavidin at room temperature for 1 hour. The intensity of signals was detected directly from the membranes using a chemiluminescene imaging system (Luminocapture AE6955; Atto Co., Tokyo, Japan). Exposure times ranged from 30 seconds to 2 minutes. Chemiluminescence was quantified with Atto imaging and analysis software. HRP-conjugated antibody served as a positive control at six spots and was also used to identify the membrane orientation. For each spot, the net intensity gray level was determined by substracting the background gray levels from the total raw intensity gray levels. The relative intensity levels of the cytokines were normalized with reference to the amounts of cytokines present on the positive control in each membrane on the following basis: average of the cytokine spot intensity levels/average of the positive control spot intensity levels, indicated as a percentage. Both the cytokine detection level and the within-subjects reproducibility of this array have been described in a previous report (Matsunaga et al. 2006).

### Total protein measurements in EBC, saliva, and ELF

The total protein concentrations of the saliva and ELF samples were measured with a Bradford protein assay kit (Bio-Rad, Hercules, CA, U.S.A.) (Bradford 1976). The protein concentration of EBC was measured with a Micro BCA Protein Assay kit (Pierce, Rockford, IL, U.S.A.) (Smith et al. 1985).

### Pulmonary function

Forced expiratory volume in one second (FEV_1_) and forced vital capacity (FVC) were measured with a Vitalograph Pneumotrac 6800TM (Vitarograph Co., Ennis, Ireland) according to a standard procedure (Standardization of Spirometry, 1994).

### Statistical analysis

Statistical analysis was performed using the statistical software package Stat View (Abacus Concepts, Berkley, CA). Comparisons of the characteristics between two groups (asthmatic groups and ELF group) were performed by the Student’s t-test. Comparisons of the total protein concentration between EBC and saliva were performed by the Paired t-test. Comparisons of cytokine expression levels between EBC and saliva were performed by the Wilcoxon signed-ranks test. The results were reported as means ± SE except cytokine expression levels. Each cytokine expression level was expressed as median (interquartile range (IQR)). Significance was defined as a *p* value of less than 0.05.

## Results

### Total protein concentration

The total protein concentration was detectable in all EBC, saliva, and ELF samples. The mean total protein concentrations of EBC, saliva and ELF were 4.6 ± 1.1 μg/ml, 2,398 ± 379 μg/ml and 14,111 ± 3,477 μg/ml, respectively (Table 2). The ratio of the total protein concentration between EBC, saliva, and ELF was approximately 1:500:3000.

### Comparison of the cytokine expression levels between EBC and saliva

The expression levels of each cytokine in EBC and saliva are shown in Table 3. The cytokines were lined up in the order of their expression levels in EBC with the highest level first. The cytokine order of saliva was lined up to match that of EBC. Eotaxin, eotaxin-2, granulocyte-colony stimulating factor (G -CSF) and granulocyte-macrophage colony stimulating factor (GM -CSF) showed significantly higher expression levels in EBC than in saliva (eotaxin: *p* = 0.013, eotaxin-2: *p* = 0.037, G-CSF, GM-CSF: *p* = 0.028). In contrast, the expression levels of IL-8, tissue inhibitor of metalloproteinases-2 (TIMP2), IL-1β, monocyte chemoattractant protein 1 (MCP-1), soluble TNF receptor I (sTNF-RI), intercellular adhesion molecule 1 (ICAM-1) and IL-7 were significantly higher in saliva (IL-8, TIMP2, MCP-1: *p* = 0.005, sTNF-RI, ICAM-1: *p* = 0.009, IL-1β: *p* = 0.017, IL-7: *p* = 0.047). The differences in the other cytokines between saliva and EBC were not significant.

### Comparison of the cytokine expression levels between EBC and saliva corrected by the total protein contents

**Figure 1.**
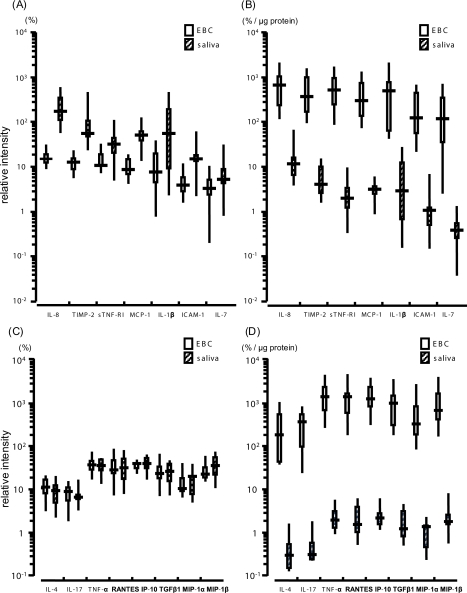
Cytokine expression levels of EBC and saliva before and after correction by the total protein content. The expression levels of seven cytokines more up-regulated in saliva than in EBC (IL-8, TIMP-2, sTNF-RI, MCP-1, IL-1β, ICAM-1 and IL-7) (Fig. 1A), and the expression levels of eight cytokines which have been reported to be more up-regulated in EBC from asthmatic patients than healthy controls (Fig. 1C) are shown. The expression levels of those cytokines corrected by the total protein content are shown (Figs. 1B and 1D). After protein correction, the expression levels of these cytokines were significantly higher in EBC than in saliva (*P* = 0.0051). Values are median (inter-quartile range).

After correction by the total protein contents, the expression levels of all cytokine in EBC were significantly higher than those in saliva. Especially, seven cytokines (IL-8, TIMP-2, sTNF-RI, MCP-1, IL-1β, ICAM-1 and IL-7), which were more up-regulated in saliva than in EBC before protein correction, also showed significantly higher expression levels in EBC than in saliva (Figs. 1A and 1B). Likewise, the corrected expression levels of the eight cytokines (IL-4, IL-17, TNF-α, RANTES, IP-10, TGF-β1, MIP-1α and MIP-1β), which had been reported to be more up-regulated in EBC from asthmatics (Matsunaga K et al. 2006), were significantly higher in EBC than in saliva (Figs. 1C and 1D).

## Discussion

In the present study, EBC and saliva were found to have different levels cytokine expression. Among the examined molecules, eotaxin, eotaxin-2, GM-CSF and G-CSF were significantly more up-regulated in EBC than in saliva, although the total protein concentration of saliva was 500 times higher than that of EBC. Corrected by the total protein contents of the EBC and saliva, all cytokine expression levels measured in this study were significantly higher in EBC than in saliva. These data suggest that the effect of salivary contamination on the cytokine assessment in EBC would be very small and that the main contributor to EBC cytokines would be ELF.

The influence of salivary contamination on EBC has been examined in several studies (Griese et al. 2002; Gaber et al. 2006; Horvath et al. 2005; Mutlu et al. 2001; Effros et al. 2002). Amylase activity was once recommended to be measured routinely for monitoring the salivary contamination. However, amylase is not specific to saliva and at present the routine measurement of amylase activity is not recommended. The electrolyte ratios in saliva have been reported to differ from those in EBC (Effros et al. 2002). Further, EBC has been shown to contain proteins not present in saliva using two-dimensional gel electrophoresis (Griese et al. 2002). Thus, saliva has not been considered to be a major contributor to EBC. However, the influence of salivary contamination on the assessment of inflammatory molecules in EBC has not been fully investigated. Although Simpson JL et al. measured the IL-8 levels in EBC and saliva samples from asthmatic patients, IL-8 was not measurable in all EBC samples and the detection level was almost near the detection limit of the assay. Furthermore, the salivary influence on the IL-8 level in EBC was not assessed (Simpson et al. 2005). The salivary influence on EBC cytokines has not been adequately clarified until now.

In the present study, the expression levels of several cytokines were found to be more up-regulated in EBC than in saliva. As mentioned above, when corrected by the total protein contents, the corrected cytokine expression levels of all cytokines were significantly higher in EBC than in saliva. This suggests that EBC and saliva might have different cytokine properties and the salivary influence on EBC cytokine might be negligibly small. A cytokine protein array is a semiquantitative assay and the precise contribution of saliva is still unclear. However, it has been shown that the relative levels obtained by the protein array correlated well with the actual levels obtained by quantitative assays in several previous reports (Huang et al. 2002a; Huang et al. 2002b; Lin et al. 2003; Turtinen et al. 2004) and our results should therefore be conducive to the standardization of EBC collection.

Protein has been used as a dilution marker for EBC and BAL assessment (Jackson et al. 2007). As described in that report, protein may not be an ideal marker as a dilution parameter, but the protein can be readily measured with a small sample volume. In the present study, the sample volume of ELF was too low (10∼20 μl) and, therefore, we used it as a dilution marker of EBC. Although various markers such as conductivity, electrolyte and glucose have been measured to estimate the dilution of EBC, currently there is no definitive recommendation. The values of the total protein concentration of EBC from the present study groups were similar to those of previous reports (Effros et al. 2002; Effros et al. 2003). However, the values may be affected by the collecting device or breath condenser coatings. The concentrations of cysteinyl-Leukotriens and eotaxin were significantly higher in EBC collected by ECoScreen than in that by RTube (Soyer et al. 2006). The albumin and 8-isoprostane concentrations of EBC differed among five condenser coatings (Alfaro et al. 2007). Significant differences in the thromboxane A(2) (TXA2) concentration of EBC and the detection rate of TXA2 in EBC were found between enzyme immunoassay and radioimmunoassay (Huszar et al. 2005). Consider these technical effects on the EBC protein levels and further study will be needed for the standardization of EBC assessment.

The lower airways have been considered as the dominant source of non-volatile substances in EBC. The dilution of EBC has been estimated as approximately 1:2000∼1:20000 by comparing the conductivity and electrolyte concentration in EBC with those in plasma (Effros et al. 2002; Effros et al. 2003). The glucose concentration has been measured in EBC to estimate the glucose concentration in respiratory fluid, although the glucose level in EBC is affected by the underlying disease such as diabetes and lung disease (Baker et al. 2007). However, a direct comparison of the solute concentrations between EBC and the lower airway ELF has not been performed yet. Our report is the first study in which a direct comparison between EBC and ELF were performed although samples were obtained from different groups. In the present study, the mean total protein concentration of ELF was found to be approximately 3,000 times higher than that of EBC, which suggests that the dilution of EBC could be estimated as 1:3000. In addition, the mean total protein concentration of ELF was six times higher than that of saliva. This indicates that the extent to which saliva would contribute to EBC should be very small compared with ELF. Our result supports the hypothesis that the dominant source of non-volatile substances in EBC is the lower airway (Horvath et al. 2005) and previous reports that inflammatory molecule analysis of EBC is useful for monitoring the asthmatic airway condition (Shaid et al. 2002; Ko et al. 2006; Matsunaga et al. 2006; Simpson et al. 2005).

In this study, we used the mean values of the total protein concentrations of EBC and ELF from different groups to roughly estimate the dilution of EBC and the expression levels of ELF, although there are some methodological problems. We didn’t perform a bronchoscopic examination for asthmatic patients because such an examination could induce an asthma attack. Although the actual cytokine expression levels of ELF should have been measured, the sample volume of ELF was too low to be applied for a cytokine array assay. Furthermore, biomarkers such as 8-isoprostane, NOx, and H_2_O_2_ in EBC were reported to be not correlated with those in bronchoalveolar lavage (BAL) (Jackson et al. 2007). However, BAL is generally accepted as a sampling of only the smaller airways and alveoli. In this study, ELF was obtained from the larger airway (the right or left main bronchus). We previously reported that the albumin concentration of ELF obtained from COPD patients showed a strong correlation with %FEV_1_ and there was no significant difference in the albumin levels according to the smoking status and age (Minakata et al. 2005). This suggests that the origin of EBC might be the central airways. Although subjects of ELF group in this study were significantly older than those of the asthmatic group, there was no difference in the degree of airflow limitation between the asthma and ELF groups. Thus, the mean total protein concentrations of ELF from the ELF group can be expected to be similar to those of the asthmatic group. Accordingly, we expect our result will be useful for future EBC studies despite its rough estimation.

In conclusion, the cytokine expression patterns of EBC and saliva were found to be different and the source of cytokines in EBC should be those from the lower airway. This suggests that the salivary influence on the assessment of EBC cytokines would be negligibly small.

## Figures and Tables

**Table 1.** Characteristics of study subjects.

	**Asthmatics**	**ELF group**	**p value**
Subject number	10	10	
Age(yr)	44.6 ± 5.4	63.1 ± 5.3	0.025
Sex			
female/male	5/5	1/9	
FVC(L)	3.35 ± 0.16	3.51 ± 0.29	0.824
FEV_1_ (L)	2.48 ± 0.18	2.55 ± 0.25	0.647
FEV_1_%(%)	73.9 ± 3.6	74.2 ± 5.1	0.960
%FEV_1_(%)	84.5 ± 6.5	87.9 ± 5.2	0.691

FEV_1_: forced expiratory volume in one second; FVC: forced vital capacity values are means ±SE.

**Table 2.** Total protein concetration of each sample.

	**EBC**	**Saliva**	**ELF**
total protein concentration (μg/ml)	4.6 ± 1.1	2,398 ± 379*	14,111 ± 3,477^**†^

The samples of both EBC and saliva were obtained from the same asthmatic patients. ELF samples were obtained from another subject group who had undergone bronchoscopic examination. The ratio of the total protein concentration between EBC, saliva, and ELF was approximately 1:500:3000.

* *P* = 0.0001 compared with the value of EBC

** *P* = 0.0007 compared with the value of EBC

† *P* = 0.0036 compared with the value of saliva. Values are means ± SE.

**Table 3.** Each cytokine expression level in ebc and saliva.

**Cytokines**	**EBC(%)**	**Saliva(%)**	**p value**	**Cytokines**	**EBC(%)**	**Saliva(%)**	**p value**
TNF-β	41.4 (36.6–51.6)	43.8 (33.8–52.6)	0.721	IL-16	10.3 (8.5–21.9)	11.4 (3.5–17.2)	0.386
IP-10	37.0 (28.0–37.9)	36.6 (28.2–50.1)	0.575	M-CSF	10.3 (8.1–14.2)	12.4 (8.9–21.8)	0.139
TNF-α	34.3 (25.9–43.8)	32.9 (24.8–46.3)	0.333	GCSF	9.9 (5.2–12.1)	5.6 (2.3–6.9)	0.028
RANTES	25.8 (21.4–44.5)	29.7 (15.9–49.2)	0.575	IL-2	9.9 (8.0–26.9)	10.9 (4.4–22.1)	0.799
TGF-β1	21.8 (16.3–31.5)	24.0 (14.3–36.8)	0.959	MIP-1α	9.6 (8.2–16.8)	18.1 (7.1–19.5)	0.575
MIP-1β	20.8 (16.8–30.3)	32.9 (19.6–52.4)	0.241	MCP-1	8.7 (6.8–15.1)	51.2 (38.7–62.5)	0.005
IL-15	15.9 (6.2–26.6)	11.5 (5.4–14.3)	0.114	IL-17	8.2 (5.1–10.5)	6.1 (5.4–7.1)	0.386
EOTAXIN2	15.5 (14.5–23.0)	12.4 (5.4–20.3)	0.037	IL-1β	7.9 (4.6–20.9)	56.3 (9.3–199.6)	0.017
IL-8	15.5 (12.2–19.7)	174.8 (108.1–358.8)	0.005	IL-6	6.9 (4.4–9.4)	5.9 (2.4–7.9)	0.386
PDGF-BB	14.0 (10.6–17.5)	16.8 (11.3–23.2)	0.386	MIG	6.7 (5.0–6.9)	8.7 (4.4–13.1)	0.333
IL-6sR	13.3 (7.6–14.6)	11.9 (9.2–15.9)	0.799	I-309	6.5 (3.0–8.0)	4.6 (1.3–7.3)	0.241
EOTAXIN	13.2 (10.5–21.8)	10.1 (5.4–17.8)	0.013	IL-12p40	4.8 (3.4–7.2)	8.1 (2.9–12.3)	0.799
sTNF R II	13.1 (10.5–14.1)	16.2 (13.5–19.0)	0.386	IFN-γ	4.7 (4.2–11.3)	5.1 (1.4–6.7)	0.093
TIMP-2	12.9 (9.0–15.7)	56.7 (48.2–110.4)	0.005	GM-CSF	4.6 (3.2–5.6)	2.3 (1.8–4.8)	0.028
IL-10	12.5 (10.0–20.6)	18.6 (13.1–25.0)	0.093	IL-13	4.6 (1.6–13.0)	5.4 (2.4–14.3)	0.508
MIP-1δ	12.5 (5.9–18.7)	19.3 (5.2–32.4)	0.169	MCP-2	4.1 (3.1–8.4)	5.0 (4.6–9.1)	0.508
IL-3	11.5 (7.0–21.5)	5.8 (3.6–13.8)	0.114	ICAM-1	4.0 (2.9–6.1)	14.9 (13.4–19.0)	0.009
sTNF-R I	11.1 (10.6–19.3)	32.6 (20.6–45.9)	0.009	IL-7	3.3 (2.3–5.4)	5.4 (4.3–9.1)	0.047
IL-1α	10.7 (7.7–18.2)	8.4 (2.3–12.6)	0.093	IL-12p70	3.0 (2.0–5.4)	4.8 (0.3–9.7)	0.721
IL-4	10.4 (7.4–15.9)	8.7 (4.5–11.7)	0.241	IL-11	1.8 (1.1–3.6)	2.8 (2.1–6.9)	0.333

Cytokines are lined up in the order of their expression levels in EBC with the highest first. The cytokine order of saliva is lined up to match that of EBC.

**Abbreviations:** Mig, Monokine induced by IFN-γ; IP-10, IFN-γ–in-ducible protein 10; MIP, Macrophage inflammatory protein; IL-6sR, IL-6 soluble receptor; M-CSF, macrophage colony-stimulating factor; PDGF, platelet-derived growth factor. Values are median (interquartile range).
